# A review of the non-metallic *Osmia* (Melanosmia) found in North America, with additional notes on palearctic *Melanosmia* (Hymenoptera, Megachilidae)

**DOI:** 10.3897/zookeys.60.484

**Published:** 2010-10-07

**Authors:** Molly G. Rightmyer, Terry Griswold, Michael S. Arduser

**Affiliations:** 1 USDA-ARS Bee Biology and Systematics Laboratory, BNR 244 UMC 5310, Utah State University, Logan UT, 84322-5310 USA; 2 Missouri Department of Conservation, 2360 Highway D, St. Charles MO 63304

**Keywords:** Bee, Apoidea, Megachilinae, Osmiini

## Abstract

We review the six species of non-metallic Osmia (Melanosmia) found in North America, including the description of two new species found in Canada and the northern United States: Osmia (Melanosmia) aquilonaria **sp. n.**, and Osmia (Melanosmia) nearctica **sp. n.**, respectively belonging to the inermis and xanthomelana species groups. We additionally provide keys to the non-metallic Melanosmia found in North America, and update keys to the palearctic Melanosmia based on study of the type specimens of Osmia disjuncta Tkalců, Osmia ephippiata Smith, Osmia ishikawai Hirashima, and Osmia pamirensis Gussakovskij.

## Introduction

This is a treatment of the members of the subgenus Melanosmia (*sensu* Michener 2007) that lack any metallic blue or green coloration on the integument of the mesosoma and metasoma (the head also usually lacks metallic blue or green coloration, although there is a hint of such color in the head of some males). For this reason, some workers informally refer to such species as the “dark Osmia.” As treated herein, the group roughly corresponds to Melanosmia s. str. as used by Sandhouse (1939), excluding Osmia bucephala Cresson (1864), which we have excluded based on the slight metallic blue sheen found throughout the body of this species. It is currently unknown if the “dark Osmia” as a group has any phylogenetic standing; our decision to assemble and give taxonomic treatment to these species is based solely on the ease of distinguishing its members. In addition, certain species in this group are distributed in both the Eastern and Western Hemispheres, without, as far as we know, having been introduced by human activity. Other holarctic species of Osmia are rare and known to be human introductions. With few exceptions, most non-metallic Melanosmia are distributed in northern temperate to borealalpine regions, which possibly facilitated the widespread distribution of some species via Beringia.

As currently understood, the “dark Osmia” group found in North America is comprised of four holarctic species plus two new species apparently restricted to North America, for a total of six species. In addition, some (but not all) females of Osmia(Melanosmia) tersula Cockerell (1912) mostly lack a metallic blue or green sheen to their integument, and are therefore included in the key to non-metallic Melanosmia below.

Given the holarctic distribution of many non-metallic Melanosmia, there is a possibility that the two new North American species described herein are already known from the Palearctic. In order to avoid producing new junior synonyms, it is necessary to understand the 20 species of Melanosmia known from the Palearctic (Ungricht et al. 2008; Müller 2010) (Table 1). Unfortunately, not all species are well described in the literature and the two species described by Wu (Osmia nigroscopula Wu, 1982 and Osmia jilinensis Wu, 2004) are unknown to us although we attempted to borrow the holotype material.

Of the remaining 18 species of palearctic Melanosmia, three species (Osmia alticola Benoist 1922, Osmia maritima Friese 1885, and Osmia xanthomelana Kirby 1802) were placed in the xanthomelana species group by Tkalců (1983) due to the shared swollen gonoforceps of the males and the shining propodeal triangle (metapostnotum) of the females. Tkalců (1983) recognized a second group of palearctic Melanosmia, the inermis species group, whose members have relatively thin gonoforceps in males and dull posterior surface of the propodeum and propodeal triangle in females. The species that have been explicitly placed in the inermis species group (Tkalců 1983; Müller 2002, 2010) are as follows: Osmia inermis (Zetterstedt 1838); Osmia laticeps Thomson 1872; Osmia parietina Curtis 1828; Osmia pilicornis Smith 1846; Osmia steinmanni Müller 2002; Osmia svenssoni Tkalců 1983; and Osmia uncinata Gerstäcker 1869. Osmia disjuncta Tkalců 1995, was originally described as closely related to Osmia parietina and Osmia laticeps (as Osmia hyperborea), and upon examination of the female holotype we conclude that it also belongs to the inermis species group.

Müller (2010) segregated Osmia nigriventris (Zetterstedt 1838) into a third species group; this classification scheme highlights some unusual characteristics of Osmia nigriventris (e.g., the extremely swollen clypeal margin of the female and laterally reflexed posterior terga of the male). These unusual features are shared with the North American species Osmia bucephala, a species that has further apomorphies that have obscured its taxonomic placement within Osmia.

The remaining species of palearctic Melanosmia have received less treatment in recent revisions and are thus considerably less well-known. They are Osmia ephippiata Smith, 1879, Osmia ishikawai Hirashima, 1973, Osmia melanota Morawitz, 1888, Osmia pamirensis Gussakovskij 1930, Osmia recta Pérez, 1902, and Osmia thoracica Radoszkowski, 1874. Of these, we were able to exclude Osmia melanota from consideration as a possible component of the North American fauna due to the original description of the type material as dark metallic blue (Morawitz 1888, p. 243, 244). Osmia recta can also be excluded due to the fact that Pérez (1902, p. 63) described the holotype male as having two submedial tufts of long, blackish-grey hairs on T3 and T4; in addition, the species’ known distribution is in Algeria, quite unlike the more northern distribution of the known holarctic species.

Of the palearctic Melanosmia not treated by Tkalců (1983, 1995) or Müller (2002), we have seen the holotype specimens of Osmia ephippiata and Osmia ishikawai. We have also examined a female syntype of Osmia pamirensis, and one of us (TG) has additionally seen the type series of two males and nine females of Osmia pamirensis at the Russian Academy, St. Petersburg in 1984 (although, according to notes made by TG at the time of his visit, the males of the type series appear not to be conspecific). Tkalců (1983) did not treat females of Osmia laticeps (as Osmia hyperborea) in his revision of palearctic Melanosmia; the female of this species was diagnosed by Haeseler (1999). Herein we give further description of the female of Osmia laticeps and include it in an updated key to palearctic Melanosmia based on Tkalců (1983, 1995) and Müller (2002).

**Table 1.** Palearctic species of Osmia (Melanosmia), with holarctic species in bold.

Osmia (Melanosmia) species	Species group	Integument type†	Examined material‡
1. Osmia alticola Benoist	xanthomelana, (based on Tkalců, 1983)	faint metallic sheen in ♂ (A. Müller, pers. comm.)	None
2. Osmia disjuncta Tkalců	inermis	non-metallic	♀ Holotype and ♀, ♂ paratypes
3. Osmia ephippiata Smith§	xanthomelana? (based on body size and mandible)	non-metallic	♀ Holotype
4. Osmia inermis (Zetterstedt)	inermis	non-metallic	♀, ♂ non-type specimens
5. Osmia ishikawai Hirashima	inermis	non-metallic	♀ Holotype
6. Osmia jilinensis Wu	?	non-metallic (based on Wu 2005)	None
7. Osmia laticeps Thomson	inermis	non-metallic	♀, ♂ non-type specimens
8. Osmia maritima Friese	xanthomelana	non-metallic	♀, ♂ non-type specimens
9. Osmia melanota Morawitz	?	metallic (based on Morawitz 1888)	None
10. Osmia nigriventris (Zetterstedt)	nigriventris	non-metallic	♀ specimens compared with syntypes of Osmia corticalis by TG; ♀, ♂ non-type specimens
11. Osmia nigroscopula Wu	?	non-metallic (based on Wu 2005)	None
12. Osmia pamirensis Gussakovskij	xanthomelana	slight metallic sheen	♀ Syntype
13. Osmia parietina Curtis	inermis	slight metallic sheen	♀, ♂ non-type specimens
14. Osmia pilicornis Smith	inermis	faint metallic sheen in ♂	♀, ♂ specimens compared with syntypes by TG
15. Osmia recta Peréz	?	?	None
16. Osmia steinmanni Müller	inermis	faint metallic sheen in ♂	♀, ♂ Paratypes
17. Osmia svenssoni Tkalců	inermis	non-metallic	♀, ♂ Paratypes
18. Osmia thoracica Radoszkowski	xanthomelana?	non-metallic?	♀ non-type specimen |
19. Osmia uncinata Gerstäcker	inermis	non-metallic	♀, ♂ specimens compared with syntypes by TG; ♀, ♂ non-type specimens
20. Osmia xanthomelana (Kirby)	xanthomelana	non-metallic	♀ specimen compared with syntypes by TG; ♀, ♂ non-type specimens

† “Integument type” refers to the presence or absence of metallic blue or green coloration. ‡ “Examined material” gives details of material examined for this study; information gathered from the literature or personal communication, and not based on examined material, is noted with citations in the table. § Distinctive characters of the Osmia ephippiata holotype are the following: mesepisternum and metasomal terga (including what is visible on T1) with entirely black hairs; mandible with parallel outer and condylar ridges; mandible with third tooth partially obscured by debris, but apparently more or less triangular between second and fourth teeth. The holotype of Osmia ephippiata has the metasoma glued to the mesosoma, so it is not currently possible to determine the sculpturing of its propodeum or declining anterior surface of T1; also, part of the dorsal surface of T1 is obscured by the glue. However, Tkalců (1983: 155) was able to examine the anterior surface of T1 in 1965, and he commented that it is distinctly shining. This observation was confirmed by George Else in 1977 (ibid). | Female specimen of Osmia thoracica Radoszkowsi from Hakkari, Turkey, identified by K. Warncke.

## Materials and methods

The morphological terminology used herein follows that proposed by Michener (2007), with the exception of the following terms: flagellar segment instead of flagellomere, and basitarsal segment instead of basitarsus; in addition, we follow sculpture and punctation terminology proposed by Harris (1979). Mandibular teeth are numbered from ventral-most tooth to dorsal-most tooth. Thus, the ventral-most tooth is the first tooth and the next ventral-most tooth is the second. In the species treated herein, between the second and dorsal-most tooth is a smaller, slightly more inset, cutting edge extending from the dorsal-most tooth, here called the third tooth. The dorsal-most tooth is the fourth tooth.

The following morphological abbreviations are used: flagellar segment (F), metasomal tergum (T), metasomal sternum (S), and ocellar diameter (OD). Measurements are given for the holotype specimen, with the observed range from other specimens following in parentheses.

Full label data are given for all specimens of new species.  Label data of examined material for the remaining species were summarized at the county level or its equivalent, along with date, floral record, and altitude (if given). The following abbreviations are used for specimen repositories, with individuals associated with those repositories following in parentheses:

BoulderUniversity of Colorado, Boulder CO (V. Scott)

CorvallisOregon State University, Corvallis OR (C. Marshall)

DavisUniversity of California, Davis CA (S. Heydon)

LoganUSDA Bee Biology and Systematics Laboratory, Logan UT (T. Griswold, H. Ikerd)

Logan-TGPersonal collection of T. Griswold, Logan UT

MoscowUniversity of Idaho, Moscow ID (J. B. Johnson, F. M. Merickel)

New YorkAmerican Museum of Natural History, New York NY (J. S. Ascher, J. G. Rozen, Jr.)

OttawaCanadian National Collection, Ottawa (L. Dumouchel, P. LeClair)

San FranciscoCalifornia Academy of Sciences, San Francisco CA (W. Pulawski, R. Zuparko)

St. CharlesMissouri Department of Conservation, St. Charles MO (M. Arduser)

TorontoRoyal Ontario Museum, Toronto Ontario (B. Hubley)

UppsalaUppsala University, Sweden (B. G. Svensson)

VictoriaRoyal British Columbia Museum, Victoria B. C. (R. Cannings)

Specimens were examined and measured using a Leica MZ12 dissection microscope and ocular micrometer. Photomicrographs were taken using a Keyence Digital Imaging System.

## Key to North American Females of Non-Metallic Osmia (Melanosmia)

[Modified from Tkalců 1983, 1995]

**Table:** 

1.	Apical margin of clypeus strongly thickened (Figs 11, 12)	Osmia nigriventris (Zetterstedt)
–	Apical margin of clypeus more or less flat (Figs 4, 6)	2
2.	Ventral margin of mandible with distinct tooth (Fig. 52) (propodeal triangle strongly granulose, Fig. 56; mandible with apical margin about a third wider than median width, Fig. 4)	Osmia inermis (Zetterstedt)
–	Ventral margin of mandible sometimes slightly swollen medially, but lacking distinct tooth	3
3.	Propodeal triangle strongly granulose (Figs 16, 56)	4
–	Propodeal triangle with more or less shining ventral area (Figs 36, 57) (mandible with apical width 1.5 times greater than median width) [xanthomelana species group]	6
4.	T2–T3 with apical impunctate bands nearly one-third of postgradular width	Osmia tersula Cockerell (western form)
–	T2–T3 with apical impunctate bands lacking or about one-fifth of postgradular width [inermis species group]	5
5.	Mandible with third tooth broad, neither strongly protruding nor strongly separated from fourth tooth (Fig. 6), with condylar and outer ridges strongly converging apically (Fig. 5); mesepisternum with white to pale yellow hairs; T1 dorsal surface with distinct punctures (Fig. 58)	Osmia laticeps Thomson
–	Mandible with third tooth symmetrically triangular, broadly separated from both fourth and second teeth by acute-angled and broad indentation (Fig. 2), with condylar and outer ridges nearly parallel to weakly converging apically (Fig. 1); mesepisternum with hairs predominantly blackish, only on its most anterior and dorsal part with narrow zone bright yellowish-brown; T1 dorsal surface granulose/papillate, with punctures less strongly impressed (Fig. 17)	Osmia aquilonaria Rightmyer, Griswold, & Arduser, sp. n.
6.	Mandible with third tooth in same plane as second and fourth teeth, lacking distinct carina separating it from second and fourth teeth (Fig. 10); outer hind tibial spur weakly curved apically; clypeus with apical truncate process with distinct lateral angle, margin lateral of truncation distinctly stairstepped (Fig. 35); hair fringe of galea in dorsal view shorter than width of malar space at mandibular condyle	Osmia nearctica Rightmyer, Griswold, & Arduser, sp. n.
–	Mandible with third tooth distinctly recessed between second and fourth teeth, with carina separating it from second and fourth teeth (Fig. 8); outer hind tibial spur strongly curved apically; clypeus with apical truncation not distinctly angled laterally, margin lateral of truncation sinuate but not distinctly stairstepped (Fig. 55); hair fringe of galea in dorsal view as long as width of malar space at mandibular condyle	Osmia maritima Friese

## Key to North American Males of Non-Metallic Osmia (Melanosmia)

[Modified from Tkalců 1983, 1995; and Müller 2002]

**Table:** 

1.	T5 and T6 with apicolateral angles strongly reflexed laterally (Fig. 53)	Osmia nigriventris (Zetterstedt)
–	T5 and T6 with apicolateral angles not or only weakly reflexed laterally	2
2.	Outer margin of gonoforceps preapically with semicircular widening, at this point gonoforceps appearing nearly twice as broad as its narrowest width (Figs 45, 49, 50, 64) [xanthomelana species group]	3
–	Outer margin of gonoforceps preapically not or only weakly widened, at this point gonoforceps at most little broader than at its narrowest width (Figs 26, 30, 31) [inermis species group]	4
3.	Flagellar segments on ventral surface with hairs microscopic; S2 with midapical margin not emarginate	Osmia nearctica Rightmyer, Griswold, & Arduser, sp. n.
–	Flagellar segments on ventral surface with sparse hairs, their length about half the flagellar segment width; S2 with midapical edge weakly emarginate	Osmia maritima Friese (based on observed palearctic material only)
4.	S4 with hooked bristles both along apical margin and on premarginal area, along apical margin the bristles oriented horizontally and on premarginal area directed increasingly vertically (Figs 24, 25)	Osmia aquilonaria Rightmyer, Griswold, & Arduser, sp. n.
–	S4 with midapical hairs unmodified, without hooked bristles (Figs 60, 61)	5
5.	S4 with apical margin truncate, medially with strong emargination and distinct, rounded lobes lateral to emargination (Fig. 60); declining basal portion of T1 densely shagreened, only with a silky luster	Osmia inermis (Zetterstedt)
–	S4 with apical margin more or less evenly convex, lacking strong emargination at midpoint and distinct sublateral lobes (Fig. 61); declining basal portion of T1 shining, at most superficially shagreened in small areas	Osmia laticeps Thomson

## Key to Eastern Hemisphere Females of Osmia (Melanosmia)

[Modified from Tkalců 1983, 1995; Haeseler 1999; and Müller 2002. Species absent from keys in the treatments cited above and for which we have seen no specimens are excluded: Osmia jilinensis, Osmia melanota, Osmia nigroscopula, and Osmia recta. (See Table 1) ].

**Table:** 

1.	Apical margin of clypeus strongly thickened	Osmia nigriventris (Zetterstedt)
–	Apical margin of clypeus more or less flat	2
2.	Propodeum shiny, propodeal triangle nearly completely polished or at least along sides with shiny area. Body length at least 11 mm [xanthomelana species group]	3
–	Propodeum and propodeal triangle completely shagreened and dull; body length at most 10 mm [inermis species group]	6
3.	T2 with impunctate apical margin medially rather broad (ca. length of F10); T4 polished (excluding the shagreened, impunctate apical margin)	4
–	T2 with impunctate apical margin narrower; T4 more or less shagreened throughout	5
4.	Integument with weak metallic sheen; mandible with rather weak, nearly absent, carina separating third tooth from second and fourth	Osmia pamirensis Gussakovskij
–	Integument lacking metallic sheen; mandible with relatively strong carina separating third tooth from second and fourth	Osmia maritima Friese, Osmia ephippiata Smith, and Osmia thoracica Radoszkowski [see Table 1 for further comments]
5.	Mandible with third tooth directed in same plane as second and fourth teeth, not set off from dorsal surface of mandible by carina; T2 with hairs medially on disc relatively long (ca. 640 micrometers), predominantly yellow-brown	Osmia xanthomelana (Kirby)
–	Mandible with third tooth directed slightly towards inner surface of mandible, set off from dorsal surface of mandible by carina extending from inner margins of second and fourth teeth; T2 with hairs medially on disc relatively short (ca. 400 micrometers), entirely black	Osmia alticola Benoist
6.	Declining basal portion of T1 densely shagreened, only with a silky luster	7
–	Declining basal portion of T1 shining, at most superficially shagreened in small areas	10
7.	Mandible with third tooth broad, not strongly separated from fourth tooth; forewing with veins cu-v and M intersecting vein M+Cu at the same place	8
–	Mandible with third tooth more triangular; forewing with cu-v intersecting vein M+Cu at a point distal to that of M	9
8.	Mandible on its inferior inner margin with prominent, asymmetrically triangular tooth; third tooth nearly filling entire space between second and fourth teeth; clypeus in profile only moderately convex	Osmia inermis (Zetterstedt) and Osmia ishikawai Hirashima
–	Mandible on its inferior inner margin nearly straight, without tooth; third tooth separated from second by indentation nearly one-third of entire space between second and fourth teeth; clypeus in profile strongly convex	Osmia disjuncta Tkalců
9.	Mandible with third tooth symmetrically triangular, broadly separated from both fourth and second teeth by acute-angled and broad indentation; mesepisternum with hairs predominantly blackish, only on its most anterior and uppermost part with narrow zone yellowish-brown; T2 with marginal zone not impressed, with punctures of disc basomedially finer, separated by up to three puncture diameters	Osmia svenssoni ­ Tkalců
–	Mandible with third tooth asymmetrically triangular, separated from second tooth by acute-angled and broad indentation, from fourth by shallow and rounded indentation; mesepisternum with hairs uniformly white to yellowish-white, sometimes with few intermixed blackish hairs; T2 with marginal zone impressed on its entire width, with punctures of disc basomedially coarser, separated by up to two puncture diameters	Osmia steinmanni Müller
10.	Distance between lateral ocellus and preoccipital margin two ocellar diameters; T2 and T3 with relatively well-defined, completely impunctate apical margin; integument with slight blue metallic sheen (especially on head and metasomal terga)	Osmia parietina Curtis
–	Distance between lateral ocellus and preoccipital margin three ocellar diameters; T2 and T3 with relatively broad apical margins with sparse, unevenly scattered punctures; integument lacking any metallic sheen	11
11.	Galea with short, completely straight hairs; mesepisternum with white to pale yellow hairs; T2 with hairs in the center of disc shorter (ca. 400 micrometers); T1 only with hairs strongly yellow-brown	12
–	Galea with much longer, incurved setae; mesepisternum with blackish hairs posteroventrally; T2 with hairs in the center of disc longer (ca. 560 micrometers); T1 and T2 with hairs strongly yellow-brown (T2 at the apical edge usually with weak admixture of black hair)	Osmia pilicornis Smith
12.	Clypeus in profile more convex, with punctures near base and in paraocular area relatively large and shallow (punctures basolaterally on clypeus distinctly larger than punctures basomedially on clypeus); forebasitarsal segment relatively long and thin (length ca. 3.5 times longer than width), length slightly longer than foretarsal segments two to five	Osmia uncinata Gerstäcker
–	Clypeus in profile less convex, with punctures near base and in paraocular area relatively small and deep (punctures basolaterally on clypeus the same size as those basomedially on clypeus); forebasitarsal segment relatively short and thick (length less than 3.0 times longer than width), length slightly shorter than foretarsal segments two to five	Osmia laticeps Thomson

## Key to Eastern Hemisphere Males of Osmia (Melanosmia)

[Modified from Tkalců 1983, 1995; Haeseler 1999; and Müller 2002. Species absent from keys in the treatments cited above and for which we have seen no specimens are excluded: Osmia ephippiata, Osmia ishikawai, Osmia jilinensis, Osmia melanota, Osmia nigroscopula, Osmia pamirensis, Osmia recta, and Osmia thoracica. (See Table 1).]

**Table:** 

1.	T5 and T6 with apicolateral angles strongly reflexed laterally	Osmia nigriventris (Zetterstedt)
–	T5 and T6 with apicolateral angles not or only weakly reflexed laterally	2
2.	Outer margin of gonoforceps preapically with semicircular widening, at this point gonoforceps appearing nearly twice as broad as its narrowest width; body length at least 11 mm [xanthomelana species group]	3
–	Outer margin of gonoforceps preapically not or only weakly widened, at this point gonoforceps at most little broader than at its narrowest width; body length under 10 mm [inermis species group]	5
3.	Flagellar segments on ventral surface with long, sparse hairs, their length about half the flagellar segment width; gonoforceps with apical tip (apical to juncture with preapical swelling) relatively flattened in lateral view	4
–	Flagellar segments on ventral surface with hairs microscopic; gonoforceps with apical tip (apical to juncture with preapical swelling) tapering to a point and not so distinctly flattened in lateral view	Osmia xanthomelana (Kirby)
4.	Gonoforceps with apical tip (apical to juncture with preapical swelling) enlarged on inner margin, concave and spoon-like in dorsal view; S2 with midapical edge weakly emarginate; S6 with median truncation moderately emarginate; hind basitarsal segment relatively narrow	Osmia maritima Friese
–	Gonoforceps with apical tip (apical to juncture with preapical swelling) more or less parallel along length, not so distinctly concave in dorsal view; S2 with midapical margin not emarginate; S6 with median truncation evenly rounded, not emarginate; hind basitarsal segment relatively broad	O. alticola Benoist
5.	S4 with midapical hairs unmodified, without hooked bristles	6
–	S4 with hooked bristles both along apical margin and on premarginal area, along apical margin the bristles oriented horizontally and on premarginal area directed increasingly vertically	7
6.	S4 with apical margin clearly forming two lobes; declining basal portion of T1 densely shagreened, only with a silky luster	Osmia inermis (Zetterstedt)
–	S4 with apical margin evenly convex; declining basal portion of T1 shining, at most superficially shagreened in small areas	Osmia laticeps Thomson
7.	Flagellar segments on ventral surface with conspicuous bristles with length 0.2–1.0 times width of flagellar segment	8
–	Flagellar segments on ventral surface with hairs microscopic	10
8.	Flagellar segments with bristles on the undersurface as long as the flagellar segment width; S6 with median truncation narrow, distally covered with knobbed hairs	Osmia pilicornis Smith
–	Flagellar segments with bristles on the undersurface only 0.2 times flagellar segment width; S6 with median truncation relatively broad, without knobbed hairs	9
9.	Vertex of head in frontal view relatively strongly ascending, with outline of head thus more quadrangular; T2–T4 with marginal zone weakly impressed, densely shagreened at least on basal half; T2–T4 with hairs bright yellow-brown; S3 with midapical emargination one-fifth as deep as broad; S6 with midapical truncation more than one-half as long as broad; gonoforceps with outer margin preapically distinctly widened, broader than basally on gonoforceps (Fig. 69)	Osmia svenssoni Tkalců
–	Vertex of head in frontal view relatively weakly ascending, with outline of head thus rounder; T2–T4 with marginal zone strongly impressed on its whole width, polished to superficially shagreened; T2–T4 with hairs yellowish-white; S3 with midapical emargination one-third as deep as broad; S6 with midapical truncation less than one-half as long as broad; gonoforceps with outer margin preapically not widened, about as broad at this point as basally on gonoforceps (Fig. 66)	Osmia steinmanni Müller
10.	Hind basitarsal segment relatively short and broad, the basal two-thirds with diverging edges; gonoforceps subapical width (at bend) about the same as portion of gonoforceps immediately basal to it	Osmia uncinata Gerstäcker
–	Hind basitarsal segment relatively long and slender, the basal two-thirds not diverging; gonoforceps with subapical width (at bend) slightly but distinctly broader than portion of gonoforceps immediately basal to it	11
11.	Hind basitarsal segment with tooth positioned a third from apical margin (measured along length of segment); integument with very clear greenish-blue metallic tinge	Osmia parietina Curtis
–	Hind basitarsal segment with tooth positioned ca. half way along length; integument lacking metallic tinge on mesosoma and metasoma	Osmia disjuncta Tkalců

## Taxonomy

### Osmia Melanosmia aquilonaria

Rightmyer, Griswold, & Arduser sp. n.

urn:lsid:zoobank.org:act:E61F5E16-3FD3-48A3-B6A4-F7C6649DCF37

Figs 1, 2 13 32 

#### Diagnosis.

Males of Osmia aquilonaria are most similar to the palearctic species Osmia svenssoni and Osmia steinmanni, but can be differentiated from them by the shape and pilosity of S4 and gonoforceps (See Table 2). Osmia aquilonaria males can be distinguished from all other members of the inermis species group (except Osmia svenssoni and Osmia steinmanni) by the special form of the hairs on S4 (i.e., with two patches of hooked bristles both along apical margin and on premarginal area, along apical margin the bristles oriented horizontally and on premarginal area directed increasingly vertically, Figs 24, 25).

**Figures 1–6.**
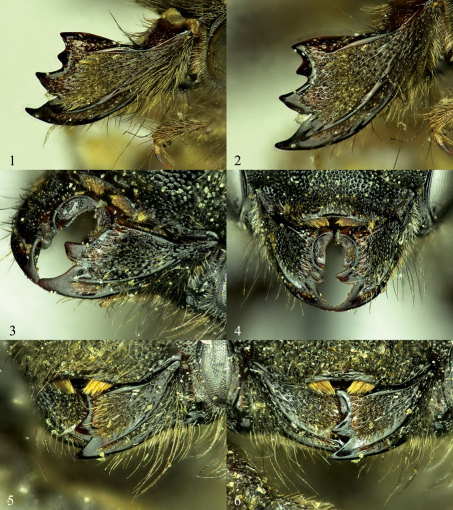
Mandibles of female Osmia, left column showing the outer and condylar ridges and overall shape of mandible, right column showing shape and placement of mandibular teeth. **1, 2** Osmia aquilonaria **3, 4** Osmia inermis **5, 6** Osmia laticeps.

Femals of Osmia aquilonaria can be distinguished from the only other nearctic member of the inermis species group, Osmia laticeps, by the more pointed third tooth and the parallel condylar and outer ridges of the mandible (Figs 1, 2; Osmia laticeps with third tooth forming cutting edge extending from fourth tooth, and with apically converging condylar and outer ridges, Figs 5, 6). Females of Osmia aquilonaria are extremely similar to those of the palearctic Osmia svenssoni, and are not readily differentiated from them other than by their respective geographic distributions.

**Table 2.** Differentiating characters of male Osmia svenssoni, Osmia steinmanni, and Osmia aquilonaria.

	Osmia svenssoni	Osmia steinmanni	Osmia aquilonaria
gonoforceps	Fig. 69; apically evenly tapering, short; preapically with outer margin widened, broader than basally; basally swollen in lateral view; hairs on preapical angle mostly on dorsal surface, relatively sparse	Fig. 66; preapically not widened, about as broad as basally; not swollen in ventral or lateral view; hairs on preapical angle mostly on dorsal surface, moderately dense	Fig. 26; apically long and slender, preapically not swollen in ventral view; basally swollen in lateral view; hairs on preapical angle mostly on lateral surface, relatively dense
S4	Fig. 70; narrower gap between brushes of hairs; brushes of hairs densely setose	Fig. 67; wider gap between brushes of hairs; brushes of hairs sparser	Figs 24, 25; wider gap between brushes of hairs, brushes of hairs densely setose
propodeal triangle	entirely granulose	lower two-thirds shining	Fig. 21; entirely granulose
head	quadrate in outline	round in outline	Fig. 20; semi-quadrate in outline
hind basitarsal segment, tooth placement (measured along length, from apical margin to basal margin)	one-fourth to one-fifth from apical margin	one-third from apical margin	Fig. 22; one-third to one-fourth from apical margin (shorter and broader than in Osmia svenssoni)

#### Description.

##### Female.

Figs 1, 2, 13–18. Total length: 8.2–11.0 mm; forewing length: 6.4–6.8 mm; length of lateral ocellus to preoccipital margin 0.7 mm; length of lateral ocellus to compound eye 0.6–0.7 mm.

###### Color:

Dark brown to brown-black, sometimes with reddish overtones especially on mouthparts, labrum, mandible, flagellar segments, legs, and apical margins of T1–T5. Wings mostly clear to weakly infuscate, except strongly infuscate along leading edge of forewing, especially along dorsal half of marginal cell.

###### Pubescence:

Clypeus below apical margin with lateral tuft of golden, medially directed hairs. Dark brown, minutely branched hairs on most of body except as follows: pale golden to white, minutely branched hairs interspersed with brown on interantennal area, vertex, posterior surface of propodeum excluding triangle, and dorsal surfaces of T1, T2, T6; almost entirely pale golden to white, minutely branched hairs on mesoscutum, mesoscutellum, and metanotum; dark-brown, simple hairs interspersed with minutely branched hairs on most of body, except simple hairs lacking on dorsal mesosoma; dark-brown, simple hairs only (no branched hairs) on all tarsi and scopa; brown, short, simple hairs evenly covering forewing. Galea and basal two labial palpal segments with hairs on lateral margins straight, 0.2–0.5 OD in length. Labrum with long hairs arranged in two curved, transverse rows, along subapical margin and approximately at midpoint, with additional fringe of shorter hairs at apical margin. Clypeus with hairs about as dense as on frons. Hypostomal area with hairs evenly distributed across area, straight to slightly wavy at apical tips, 2.5–4.0 OD in length.

###### Punctation:

Head and mesosoma with punctures nearly contiguous, more or less round, and moderately impressed except as follows: labrum mostly impunctate; clypeus with impunctate midapical truncation about length of F2 or little longer (Fig. 15); mesoscutum immediately posterior to median longitudinal sulcus with punctures separated by up to two puncture diameters; mesepisternum with punctures separated by about half a puncture diameter; hypostomal area, pronotum, and legs with punctures shallowly impressed, sometimes elongated into oval shape; tegula with punctures minute, sparse medially and posteriorly, separated by up to four or five puncture diameters; metepisternum, metanotum, and lateral and posterior surfaces of propodeum with punctures very weakly impressed, with background integument strongly granulose, dull; propodeal triangle with dorsal fourth finely areolate to lineate, lower three fourths strongly granulose, dull (Fig. 16). T1 anterior and dorsal surfaces, and T2–T5 strongly shagreened, dull, with small, sparse punctures throughout except for apical margins, these punctures with integument anterior to them slightly raised, papillate; T1–T5 apical impunctate bands with length at midpoint about 4.0–6.0 puncture diameters or little more (Figs 17, 18).

###### Structure:

Labial palpus four-segmented, second labial palpal segment ca. one-third longer than basal-most segment. Mandible with outer and condylar ridges of subequal thickness, parallel along length to very weakly converging apically (Fig. 1); apical margin with four strongly pointed teeth, third separated from second and fourth by carina, margin of third tooth forming distinct V-shape with adjacent margin of second and slightly smaller V-shape with adjacent margin of fourth, third tooth set back from second and fourth, very slightly directed inwards (Fig. 2); inner, ventral margin of mandible lacking distinct tooth, slightly diverging away from condylar ridge basally; mandible apically widened (1.3 times wider than median width), first tooth slightly longer than other teeth, length between apical tips of second and fourth teeth slightly wider than (ca. 1.2 times) apical tips of first and second teeth (Fig. 2). Clypeus with apical margin linear to moderately emarginate medially, with entire apical truncation laterally more or less contiguous with remaining lateral margin of clypeus (not forming 90 degree angle with lateral apical margin of clypeus; Fig. 15). F1 twice length of F2 or slightly more, remaining apical flagellar segments gradually increasing in length such that F10 subequal to F1 or little longer. Vertex behind lateral ocellus 2.5–3.0 OD in length. Genal width 1.5 to nearly 2.0 times that of compound eye in lateral view. Preoccipital margin rounded, not carinate. Hypostomal carina moderately high, highest at about midpoint of hypostomal area posterior to angle and sometimes forming moderate triangular projection at this point, tapering to low carina or near obsolescence at angle. Malus forming pointed apical spine, this spine more or less a continuation of nearby edge of vellum. Foretarsal segments excluding basitarsal and apical-most segments with anterior lobes slightly longer than posterior. Midtarsal segments with anterior and posterior lobes of equal width, slightly swollen; hind tarsal segments not swollen. Hind tibial spurs strongly curved at apical tips, outer spur about one fifth shorter than inner. Hind basitarsal segment with lateral margins of outer surface parallel.

##### Male.

Figs 19–32. Total length: 9.5 mm (8.2–9.5 mm); forewing length: 6.3 mm (5.9–6.3 mm); length of lateral ocellus to preoccipital margin 0.5 mm (0.4–0.5 mm); length of lateral ocellus to compound eye 0.5 mm.

###### Color:

Black to dark brown, sometimes with reddish overtones especially on mouthparts, labrum, mandible, flagellar segments, legs, and apical margins of T1–T6 and S1–S3. Wings mostly clear except weakly infuscate along leading edge of forewing, especially along dorsal half of marginal cell.

###### Pubescence:

White to golden, minutely branched hairs on body except golden to pale golden, stouter hairs on inner surfaces of tarsi, S4, and S6. Labrum covered with hairs on apical third and with hairs forming short fringe at apical margin. S2 with hairs at apical third relatively long (ca. 3.0 OD). S3 with dense, medially directed hairs filling entire emargination (hairs ca. 1.0 OD in length medially, nearly 2.0 OD laterally) (Fig. 24). S4 midapical truncation with two patches of dense, golden, distally hooked hairs, these patches of hairs medially interrupted by nearly 1.0 OD, with hairs distally meeting at midpoint, each patch along apical margin with hairs oriented horizontally and on premarginal area directed increasingly vertically (Figs 24, 25). S6 midapical truncation sparsely covered with short, distally hooked hairs arising from papillate bases (Fig. 28).

###### Punctation:

Head with punctures ovate to nearly circular, separated by one-fourth to one-half puncture diameter and deeply impressed except as follows: labrum mostly impunctate on basal two-thirds; clypeus with impunctate band along apical margin, about one-third length of F1 in length; disc of clypeus, interantennal area, and paraocular area with punctures small, ovate, and nearly contiguous (punctures mostly obscured beneath dense hairs); hypostomal area anteriorly near angle with punctures weakly, shallowly impressed. Mesosoma with punctures more or less round, nearly contiguous to separated by up to a half puncture diameter, deeply impressed except as follows: mesoscutum immediately posterior to median longitudinal sulcus with punctures separated by up to one, sometimes as much as three puncture diameters; tegula with punctures minute, sparse medially, separated by up to eight to ten puncture diameters; pronotum, metepisternum and lateral and posterior surface of propodeum strongly shagreened, with weakly, shallowly impressed, larger punctures; metanotum and propodeal triangle strongly granulose, dull (Fig. 21); propodeal triangle lineolate on dorsal fifth; legs with inner surfaces of trochanters, femora, and tibiae shining, with scattered smaller punctures. T1 with anterior surface strongly shagreened, dull; metasomal terga with dorsal surfaces excluding apical margins strongly shagreened, apical impunctate margins moderately to weakly shagreened (except T7 moderately polished). T1 dorsal surface with punctures minute, moderately distinct and well-impressed, separated from 1.0 to 3.0 puncture diameters; apical impunctate margin medially ca. 10.0 puncture diameters in length, laterally as little as 6.0 puncture diameters. T2–T7 with punctures minute, T2 with punctures separated by ca. 1.0 puncture diameter medially (sparser towards impunctate apical margin on all terga), successively posterior terga with punctures progressively becoming more widely spaced to about 3.0 puncture diameters apart on disc of T7; T2–T6 with apical impunctate margins 6.0–9.0 puncture diameters in length, T7 with apical impunctate margin 4.0–6.0 puncture diameters in length. S1–S3 with punctures weakly, shallowly impressed. S4 with integument granular, dull (Fig. 24). S5–S6 lacking distinct punctures, weakly shagreened.

###### Structure:

Mandible with outer and condylar ridges converging apically; apical margin with two teeth, upper tooth distinctly shorter and slightly wider than lower, upper tooth with inner and dorsal margins forming ca. 70–80 degree angle; inner, ventral margin of mandible weakly diverging away from condylar ridge basally. Clypeus apical margin with irregular tubercles, lacking distinct apical truncation. Flagellar segments subequal in length, except F1 about three-fourths length of F2 and F11 slightly longer than other segments. Vertex behind lateral ocellus 2.0 OD in length or nearly so. Genal width subequal that of compound eye in lateral view (slightly wider dorsally). Preoccipital margin rounded, not carinate. Hypostomal carina moderately high, gradually tapering to near obsolescence at angle, not forming distinct tooth. Malus forming small but distinct apical spine. Foretarsal segments excluding basitarsal and apical-most segments with lobes slightly, equally swollen. Mid- and hind tarsal segments not swollen. Hind tibial spurs curved at apical fifth, outer spur slightly shorter than inner. Hind basitarsal segment with lateral margins of outer surface weakly diverging apically, with strong tooth on inner margin (Fig. 22). T6 midapically with small but usually distinct emargination, forming ca. one-fourth to one-half of circle in outline (Fig. 23); T6 lateroapical margin smoothly, weakly convex, not forming distinct tooth. T7 midapically strongly emarginate, forming semicircle about as wide as deep (ca. 0.5–0.8 OD wide), with spines on either side of emargination weakly pointed, basally nearly as wide as emargination width (Fig. 23). S2 evenly convex, covering most of S3. S3 with midapical emargination relatively wide and shallow (half entire width of sternum, 1.0 OD in length, measuring only apical margin of sternum and not including basal fringe of hairs; Fig. 24). S4 midapically with wide truncation (about half width of entire sternum), medially with shallow but distinct emargination between lateral tufts of hairs (Fig. 24). S5 with apical margin evenly, strongly concave along median half of margin. S6 with strong midapical truncation, slightly less than one-third width of sternum, truncation slightly wider than deep, apical margin of truncation weakly, evenly rounded apically, sometimes with small emargination medially (Fig. 28). S8 as in Fig. 29. Gonoforceps weakly narrowed apical to subapical bend in dorsal, ventral, and lateral views (Figs 26, 27, 30–32).

**Figures 7–12.**
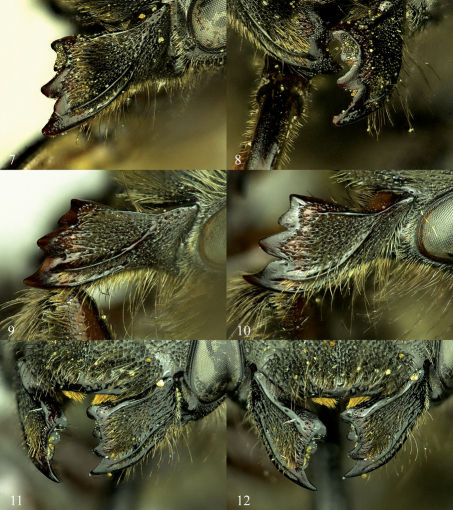
Mandibles of female Osmia, left column showing the outer and condylar ridges and overall shape of mandible, right column showing shape and placement of mandibular teeth. **7, 8** Osmia maritima **9, 10** Osmia nearctica **11, 12** Osmia nigriventris.

#### Distribution.

Alaska and Northwest Territories south to Wyoming, and east across Canada to Nova Scotia.

#### Holotype male.

“[Canada] N.W.T. [Northwest Territories] km 491, Dempster Hwy, 26.VI.80 [26 June 1980], 1000 m, Wood & Lafontaine//Osmia svenssoni Tkalcu ♂ T Griswold det 96// Holotype male Osmia aquilonaria Rightmyer, Griswold, & Arduser” (Ottawa)

**Figures 13–18.**
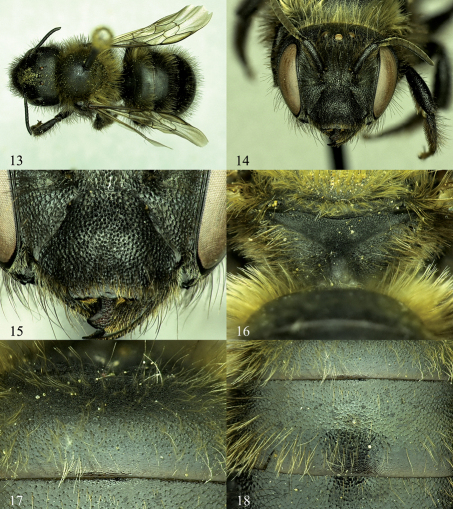
Osmia aquilonaria, female paratypes. **13** Dorsal habitus **14** Face **15** Clypeus **16** Posterior surface of propodeum and propodeal triangle **17** T1 and basal area of T2, showing surface sculpturing **18** Apical area of T1, T2, and basal area of T3.

**Figures 19–24.**
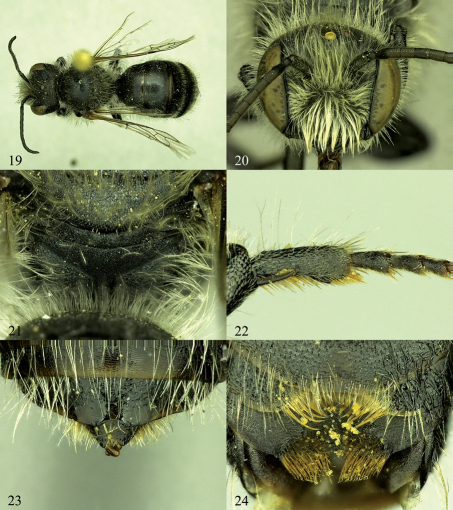
Osmia aquilonaria, male holotype. **19** Dorsal habitus **20** Face **21** Posterior surface of propodeum and propodeal triangle **22** Hind basitarsal segment, showing inner tooth **23** T5–T7 and apical tip of gonoforceps **24** S2–S4.

**Figures 25–32.**
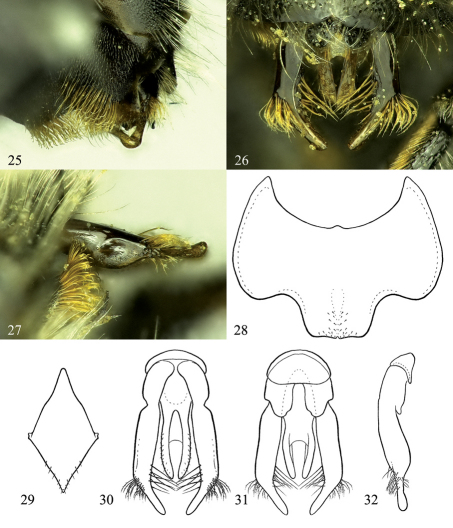
Osmia aquilonaria, male holotype and paratype. **25** Oblique view of S4 **26** Dorsal view of genital capsule **27** Lateral view of gonoforceps and S4 **28** Ventral view of S6 **29** Ventral view of S8 **30** Dorsal view of genital capsule **31** Ventral view of genital capsule **32** Lateral view of genital capsule, excluding penis valve.

#### Paratypes.

**CANADA: NORTHWEST TERRITORIES, Inuvik Region**, Aklavik, 25 June 1931, O. Bryant (1♀, Logan), 25 July 1931, 1600 ft (1♀, San Francisco); Black Mountain, SW of Aklavik, 1 August 1931, O. Bryant (1♀, 1♂, Logan); Holman, Victoria Island, 25 June 1952, B. A. Gibbard (1♀, Ottawa); **NOVA SCOTIA,** Cape Breton Highlands National Park, 60˚50'W 46˚47'N, 22 June 1983, Birch (1♀, Ottawa); **NUNAVUT, Kitikmeot Region,** Coppermine, 3 August 1951, S. D. Hicks (1♀, Ottawa); **ONTARIO, Cochrane District,** Low Bush, Lake Abitibi, 5 June 1925, N. K. Bigelow (1♀, St. Charles), 18 June 1925, N. K. Bigelow (1♀, St. Charles); **Thunder Bay District,** Silver Island, Sibley Peninsula, 18 July 1961, Rubus sp., H. E. Milliron (1♀, Ottawa); **QUEBEC, Nord-du-Québec Region,** Highway to James Bay km 66, 50˚03'N 77˚07'W, 12 June–8 August 1987, Malaise-FIT Salix bushes, L. Leblanc (1♀, Ottawa); **YUKON,** Dempster Highway km 465, 23–25 June 1980, 800 m, Wood & Lafontaine (2♀, Ottawa); **USA: ALASKA, North Slope Borough,** Cape Thompson, 29 June 1961, B. S. Heming (1♀, Ottawa); **Yukon-Koyukuk Census Area,** Kathul Mountain, Yukon River, Steppe, 4 June 1991, Arnica alpina, J. A. Bishop (1♀, Davis), 5 June 1991, Lupinus arcticus (1♀, Davis); Kathul Mountain, Yukon River, 110 km NW Eagle, Tundra, 16 June 1992, Lupinus arcticus, J. A. Bishop (1♀, Davis); **WYOMING, Fremont Co.,** Roaring Fork Mountain, Wind River Range, 29 June 1990, 11000–1200 ft, E. A. Sugden (6♀, 3♂, Logan).

#### Etymology.

The name “aquilonaria” is Latin, meaning northern or northerly, and is in reference to the northern distribution of the species in North America.

**Figures 33–38.**
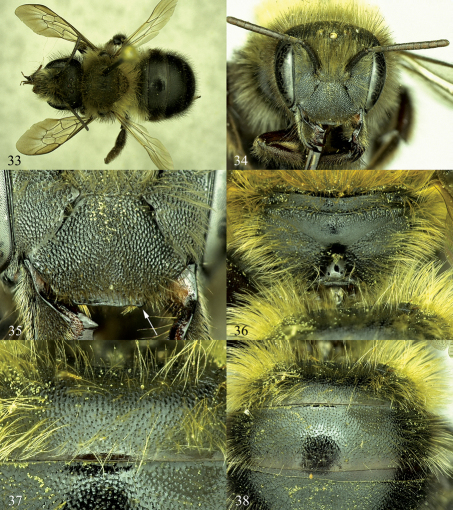
Osmia nearctica, female paratypes. **33** Dorsal habitus **34** Face **35** Clypeus **36** Posterior surface of propodeum and propodeal triangle **37** T1 and basal area of T2, showing surface sculpturing **38** Apical area of T1, T2, and basal area of T3.

**Figures 39–44.**
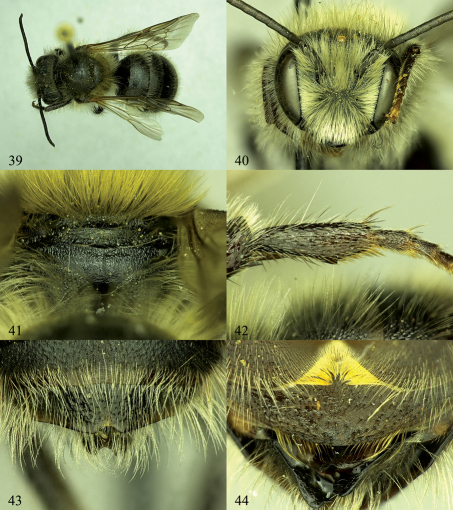
Osmia nearctica, male holotype. **39** Dorsal habitus **40** Face **41** Posterior surface of propodeum and propodeal triangle **42** Hind basitarsal segment, showing inner tooth **43** T5–T7 **44** S3 and S4.

**Figures 45–51.**
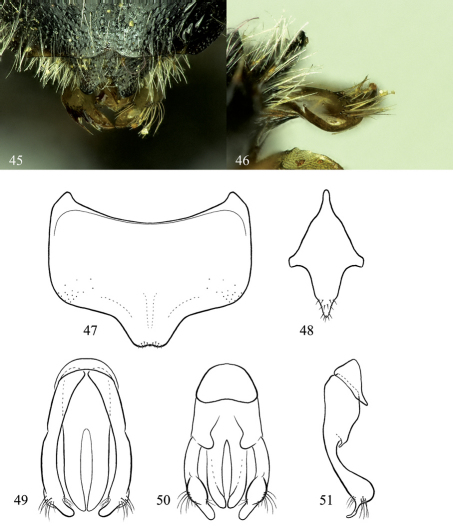
Osmia nearctica, male holotype and paratype. **45** Dorsal view of genital capsule, partially covered by T6 and T7 (T7 apical margin is ripped) **46** Lateral view of gonoforceps **47** Ventral view of S6 **48** Ventral view of S8 **49** Dorsal view of genital capsule **50** Ventral view of genital capsule **51** Lateral view of genital capsule, excluding penis valve.

### Osmia Melanosmia inermis

(Zetterstedt)

Figs 3, 4 52, 56 60

Anthophora (Osmia) inermis Zetterstedt 1838: 466 [Lectotype male: Lund]; Tkalců 1983: 153 [Lectotype designation].
Osmia globosa Cresson 1864: 36 [Holotype female: Philadelphia]; Sandhouse 1939: 34 [synonymy]; Ungricht et al. 2008: 166 [preoccupied name, not Apis globosaScopoli 1763].
Osmia vulpecula Gerstäcker 1869: 335 [Lectotype female: Berlin]; Thomson 1872: 244 [synonymy]; Tkalců 1983: 153 [Lectotype designation]
Osmia globosiformis Cockerell 1910: 311 [Holotype male: San Francisco]; Sandhouse 1939: 34 [synonymy].
Osmia (Melanosmia) inermis  (Zetterstedt); Friese 1911: 122; Sandhouse 1939: 34–35 [redescription of male and female].
Osmia (Chenosmia) inermis  (Zetterstedt); Sinha 1958: 235.

#### Diagnosis.

**Figures 52–57.**
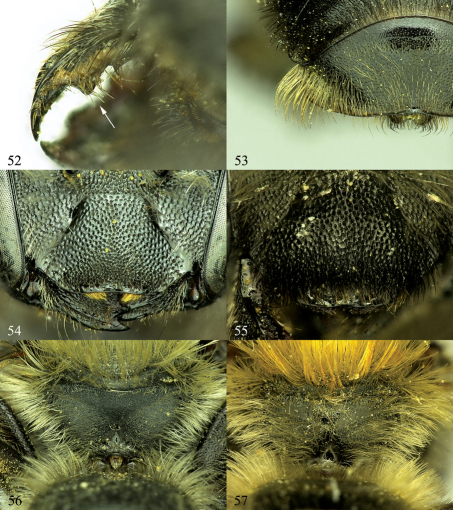
**52** Osmia inermis, female, ventral view of mandible showing tooth on inner margin of ventral surface **53** Osmia nigriventris, male, T5 and T6, showing flange on lateral margins **54** Osmia laticeps, female, clypeus with most of covering hairs removed **55** Osmia maritima, female, clypeus **56** Osmia inermis, female, posterior surface of propodeum and propodeal triangle **57** Osmia maritima, female, posterior surface of propodeum and propodeal triangle.

Females are known by the slightly acute angle or tooth midway on the ventral margin of the mandible (Fig. 52). Males can be distinguished by the form of the S4, which is strongly truncate and emarginate medially, forming distinct, rounded sublateral lobes (Fig. 60).

#### Distribution.

In the Nearctic, from Sierra Nevada of California north to British Columbia and Alaska, east through Canada to Nova Scotia and Newfoundland south in the United States to Massachusetts, Michigan, the Black Hills of South Dakota, and mountainous areas of Nevada, Utah, and Colorado. In the Palearctic, Osmia inermis is found from Spain, Italy, and Greece north to the United Kingdom, Norway, Sweden, and Finland, and east through Russia and northern China (Müller, 2010). The related, if not synonymous, species Osmia ishikawai is found in Japan.

#### Comments.

Osmia inermis has been recorded nesting under stone or in preexisting cavities in rocks and stones, with cells composed of chewed leaves (Lovell 1909; Müller 2010 and references therein). In the Palearctic, Osmia inermis is polylectic with a preference for Fabaceae (Müller 2010 and references therein); however, in Newfoundland, Canada, the species has been shown to be primarily dependent upon Ericaceae (Hicks 2009).

#### Material examined.

23 April (1♂, Boulder), 30 July 1955 (1♀, Ottawa); **CANADA: ALBERTA, Alberta’s Rockies Region**, 21 May 1915 (3♂, Ottawa, 1♂, Logan), 25 May 1892 (1♀, New York), 29 May 1922 (1♂, Ottawa), 6 July 1955 (1♀, Ottawa); **Northern Alberta Region**, 29 May 1977 (1♀, Logan); **BRITISH COLUMBIA, Central Kootenay**, 3 June 1906 (1♀, Ottawa), 9 June (1♀, Boulder); **Stikine Region**, 6 June 1955, 2200 ft (1♂, Ottawa), 17 June 1955, 2200 ft (1♀, Ottawa); **Okanagan-Similkameen District**, 21 May 1958 (1♂, Ottawa); **MANITOBA, Northern Region**, 1 July 1927 (1♀, Ottawa), 11 July 1950 (1♀, Ottawa), 29 July 1949 (1♀, Ottawa); **Parkland Region**, 26 June 1961, 2000 ft (1♀, Ottawa); **NEW BRUNSWICK, St. John Co.**, 9 June 1901 (1♀, Logan), 23 June 1901 (1♀, Ottawa); **York Co.**, 29 May 1918 (1♂, Ottawa); **NEWFOUNDLAND AND LABRADOR, Twillingate Islands**, 30 May 1951 (13♀, Ottawa); **NOVA SCOTIA, Halifax Co.**, 2 July 1914 (1♀, Ottawa); **Hants Co.**, 4 June 1931, Cornus sp. (1♂, Ottawa), 22 June 1931 (1♀, Ottawa); **ONTARIO, Kawartha Lakes**, 25 May 1964, Viola adunca (1♂, Ottawa); **Lennox and Addington Co.**, 12 May 1962, Chamaedaphne sp. (1♀, Toronto); **Rainy River District**, 2 July 1960 (1♀, Ottawa); **QUEBEC, Capitale-Nationale Region**, 17 May 1914 (2♀, Ottawa), 28 May 1916, Vaccinium sp. (2♀, Ottawa); **Nord-du-Québec Region**, 4–12 June 1987 (1♀, Ottawa), 12 June–8 July 1987 (1♀, Ottawa), 18 June 1949 (1♂, Ottawa), 14 August 1949 (1♀, Ottawa), 18 August 1949 (4♀, Ottawa), 23 August 1949 (2♀, Ottawa), 2 September 1949 (8♀, Ottawa), 3 September 1949 (1♀, Ottawa); **Outaouais Region**, 14 May 1916, Vaccinium sp. (1♀, 1♂, Ottawa); **YUKON**, 26 May 1951 (1♂, Ottawa), 31 May 1951 (2♂, Ottawa), 5 June 1951 (1♂, Ottawa), 12 June 1960, 3500 ft (1♀, Ottawa), 21 June 1949, 5200 ft (1♀, Ottawa), 2 July 1962, 3500 ft (1♀, Ottawa), 10 July 1985 (1♀, Victoria); **RUSSIA, Siberia**, 3 July 1992 (1♀, Davis), 5 July 1992 (1♀, Davis); **SWEDEN:** (1♀, Uppsala), 12–19 June 1972 (1 ♀, Uppsala), **Jönköping Co.**, 12 July 1932 (1♀, Logan); **Norrbotten Co.**, (1♂, Uppsala), 25 August 1975 (1♂, Uppsala); **USA: ALASKA, Fairbanks North Star Borough**, 19 May 1987 (1♀, Davis); **Kenai Peninsula**, 20 June 1951 (1♀, Ottawa); **CALIFORNIA, Madera Co.**, 19 July 2004, 3315 m, Phyllodoce breweri (1♂, Logan); **Mariposa Co.**, 15 June 2004, 3024 m (3♂, Logan), Phyllodoce breweri (1♂, Logan), 3215 m (1♂, Logan), 23 June 2004, 3112 m (1♂, Logan), 4 July 2004, 2860 m (1♀, Logan), 2847 m, Horkelia tridentata (1♀, Logan), 14 July 2005, 3112 m, (2♂, Logan), Phyllodoce breweri (1♂, Logan), 16 July 2004, 2944 m, Phyllodoce breweri (1♀, Logan), 14 August 2004, 3189 m, (1♀, Logan), 1 August 2005, 3189 m, (1♂, Logan); **Shasta Co.**, 30 July 1947, 7000 ft (1♀, Logan), **Tuolumne Co.**, 14 July 2004, 3049 m (1♀, Logan), 3114 m (1♂, Logan), Phyllodoce breweri (2♀, Logan), 15 July 2004, 3215 m, (3♂, Logan), Phyllodoce breweri (1♀, Logan), 17 July 2005, 3215 m (2♂, Logan), 28 July 2006, 3215 m, Arenaria kingii var. glabrescens (1♀, Logan), Eriogonum incanum (1♀, Logan), Phyllodoce breweri (1♀, Logan); **COLORADO, Boulder Co.**, 18 June 1940 (1♀, Boulder), 20 June 1940 (1♀, Boulder), 27 June 1939 (1♀, Boulder), 8 July 1940 (1♀, Boulder); **Grand Co.**, 22 June 1976 (1♀, Boulder); **Larimer Co.**, 19 June (1♀, Boulder), 25 July (1♀, Boulder); **Mesa Co.**, 10 July 1938 (1♀, Boulder); **Routt Co.**, 21 May 1964, 8500 ft, Erythronuim sp. (1♂, Boulder); **Summit Co**., 29 July 1961, 11700 (1♂, Ottawa); **IDAHO, Bear Lake Co.**, 10 August 1972 (1♀, Logan); **Lemhi Co.**, 20 July 1963 (2♀, Moscow); **MAINE, Knox Co.**, 28 May 1962, Vaccinium angustifolium (1♂, St. Charles); **MASSACHUSETTS, Barnstable Co.**, 16 May 1914 (1♂, Logan); **MICHIGAN, Alger Co.**, 23 May 1982, Vaccinium sp. (1♂, St. Charles), 29 May 1991, Vaccinium angustifolium (2♀, St. Charles); **Marquette Co.**, 25 May 1983, Vaccinium angustifolium (1♂, St. Charles), 9 June 1985, Gaylussacia sp. (1♀, St. Charles), 21 June 1984 (1♀, St. Charles); **MONTANA, Carbon Co.**, 10 July 1963, 5900 ft, Melilotus sp. (6♀, Boulder), 12 July 1963, 5900 ft, Melilotus sp. (1♀, Boulder), 28 July 1975 (1♀, Boulder); **Gallatin Co.**, 1 May 1927 (1♀, Logan), 24 June 2008 (1♂, Logan); **NEVADA, Elko Co.**, 9 July 1979 (1♂, Logan), 19 July 1975, 9500 ft (1♂, Logan-TG), 21 July 1976, 9600 ft (2♂, Logan-TG); **OREGON, Baker Co.**, 15 July 1930, 7100 ft (1♀, Corvallis); **Wallowa Co.**, 26 July 1929, 7500 ft (1♀, Corvallis); **SOUTH DAKOTA, Custer Co.**, 20 June 1955, Trifolium repens (1♀, St. Charles); **UTAH, Cache Co.**, 7 June 1962, (1♀, Logan), 18 June 1948, Wyethia sp (2♂, Logan), 30 June 1976, Penstemon leonardi (1♀, Logan), 4 July 1947, Ranunculus acriformis var. montanensis (1♀, 1♂, Logan), 5 July 1981, 8500 ft (1♀, Logan-TG), 17 July 1995, 8200–8600 ft, Penstemon sp. (2♀, Logan), 25 July 1971 (1♂, Logan), 28 July 1975, Penstemon cyananthus (1♀, Logan); 1 August 1965 (1♀, Logan), 4 August 1975, Potentilla fruticosa (1♀, Logan); **Grand Co.**, 8 June 1963 (1♂, Logan); **Sanpete Co.**, 25 June 1990, 10760 ft, Astragalus montii (1♂, Logan); Weber Co., 13 July 1950 (1♂, Logan); **WASHINGTON, King Co.** (1♀, 4♂, Boulder); **WYOMING, Big Horn Co.**, 6 August 2000, 8975 ft, Machaeranthera sp. (1♀, Boulder); **Carbon Co.**, 31 May 1972 (1♂, Boulder); **Fremont Co.**, 10 June 1955 (1♀, Logan), 29 June 1990, 11000–12000 ft (2♂, Logan); **Johnson Co.**, 22 July 1998 (1♀, Logan); **Sheridan Co.**, 26 June 1986 (1♀, Ottawa); **Sublette Co.**, 20 July 1959 (2♀, Logan); **Teton Co.**, July 1937 (1♀, Logan), 4 July 1983, 6700 ft, Hedysarum boreale (1♀, Logan), 13 July 1983, 6700 ft, Hedysarum boreale (1♀, Logan); 17 July 1983, 6700 ft, Hedysarum boreale (1♀, Logan).

### Osmia Melanosmia laticeps

Thomson

Figs 5, 6 54 58, 61

Osmia laticeps Thomson 1872: 242 [Lectotype female: Lund]; Dalla Torre 1896: 414 [synonymy with Osmia uncinata Gerstäcker]; Tkalců 1983: 154 [Lectotype designation]; Nilsson 2009: 51 [rejection of synonymy with Osmia uncinata Gerstäcker].
Osmia (Melanosmia) hyperborea Tkalců 1983: 156 [Holotype male: Uppsala]; Schwarz et al. 1996: 126 [synonymy with Osmia parietina Curtis]; Haeseler 1999: 454 [rejection of synonymy with Osmia parietina Curtis, diagnosis of female]; Nilsson 2009: 52 [synonymy with Osmia laticeps Thomson].

#### Diagnosis.

Females of Osmia laticeps are distinguished from all other North American non-metallic Osmia by the following characters of the mandible (Figs 5, 6): the apical margin is only slightly broader than the median width, the third tooth is relatively broad and not strongly separated from the fourth tooth, and the condylar and outer ridges converge apically; in addition, they are diagnosed by their strongly granulose propodeal triangle and relatively short apical impunctate bands on T2 and T3.

In the Palearctic, Osmia laticeps is most similar to Osmia uncinata. In addition to the characters mentioned in the key (above), the following characters can be used to distinguish females of the two species (most characters first noticed by Haeseler 1999): in Osmia laticeps, the clypeus has more plentiful pale hairs than black hairs, and these pale hairs are about the same length as the black hairs. In Osmia uncinata, the clypeus has nearly the same amount of black hairs as pale hairs, and the black hairs are distinctly longer than the pale hairs. The malus of the foretibia has the apical tip evenly tapering to a point in Osmia laticeps, while in Osmia uncinata the tip is slightly more blunt. The outer hind tibial spur is more strongly downcurved in Osmia uncinata than in Osmia laticeps. Additionally, the hairs of the hypostomal area are denser and more strongly incurved in Osmia laticeps than in Osmia uncinata.

In both the Nearctic and Palearctic, males are known by the non-swollen gonoforceps (outer margin preapically only weakly widened, about the same width as the gonoforceps basal and distal to this preapical point of inflection), and the relatively unmodified S4 (Fig. 61): the apical margin of S4 is evenly convex and midapically on S4 the relatively short, unmodified hairs arise from regularly-spaced tubercles (not forming distinct, sublateral tufts of apically hooked hairs).

#### Description.

##### Female.

**Figures 58–63.**
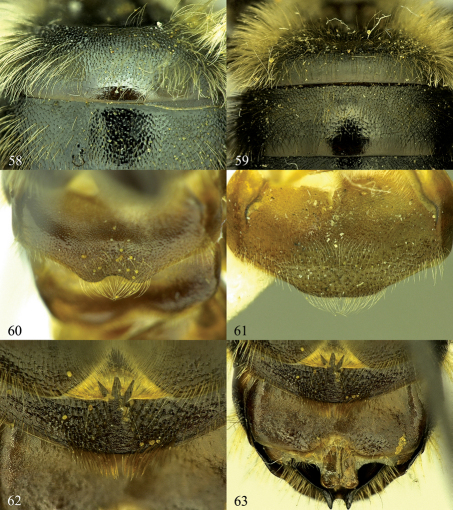
**58** Osmia laticeps, female, T1 and T2 **59** Osmia maritima, female, T1 and T2 **60** Osmia inermis, male, S4 **61** Osmia laticeps, male, S4 **62**, **63** Osmia maritima, male **62** Detailed view of S3 and S4 **63** S3–S6.

Figs 5, 6, 54, 58. Total length: 8.4–9.0 mm; forewing length: 6.0–8.1 mm; length of lateral ocellus to preoccipital margin 0.6–0.7 mm; length of lateral ocellus to compound eye 0.6–0.7 mm.

###### Color:

Dark brown to brown-black, sometimes with reddish overtones especially on mouthparts, labrum, mandible, flagellar segments, legs, and apical margins of T1–T5. Wings mostly clear to weakly infuscate, except moderately infuscate along dorsal half of marginal cell.

###### Pubescence:

Clypeus below apical margin with lateral tuft of golden, medially directed hairs. White to golden, minutely branched hairs on most of body except as follows: brown, simple hairs interspersed with pale, branched hairs on clypeus, sometimes interantennal area and near ocelli, gena ventrally and along compound eye, outer surfaces of femora and tibiae (especially on fore and midlegs); dark-brown, simple hairs only (no branched hairs) on mouthparts, labrum, inner surfaces of legs (golden on tarsi), outer surfaces of hind tibia and all tarsi, T2–T6, and scopa; brown, short, simple hairs evenly covering forewing. Galea and basal two labial palpal segments with hairs on lateral margins straight, 0.2–0.3 OD in length. Labrum with long hairs arranged in two curved, transverse rows, along subapical margin and approximately at midpoint, with additional fringe of minute hairs at apical margin. Clypeus with hairs about as dense as on frons, midapically with some hairs slightly curved at apical tips. Hypostomal area with straight hairs evenly distributed across most of area, 2.0–3.0 OD in length.

###### Punctation:

Head and mesosoma with punctures nearly contiguous, more or less round, and moderately impressed except as follows: labrum mostly impunctate except near fringes of hairs; clypeus with impunctate midapical truncation about length of F2 or little longer (Fig. 54); mesoscutum immediately posterior to median longitudinal sulcus with punctures separated by up to a puncture diameter; mesepisternum with punctures separated by about half a puncture diameter; metepisternum with punctures less distinct, separated by up to a puncture diameter; hypostomal area anteriorly near angle, posterior half of gena, and legs with punctures shallowly impressed, sometimes elongated into oval shape; tegula with punctures minute, sparse medially and posteriorly, separated by up to four puncture diameters (up to six puncture diameters in some specimens); pronotum, metanotum, and lateral and posterior surfaces of propodeum with punctures less distinctly impressed and background integument weakly shagreened; propodeal triangle with dorsal fourth reticulate to lineate, lower three fourths strongly shagreened, dull. T1 anterior surface moderately shagreened, weakly shining, with scattered, sparse, minute punctures throughout; T1–T3 dorsal surfaces weakly shagreened, shining, with small punctures nearly contiguous to separated by 2.0 puncture diameters on basal three-fourths, minute and much more sparsely spaced on apical fourth (4.0–6.0 puncture diameters apart), apical margins with narrow region entirely impunctate (T1 with apical impunctate margin polished, ca. 5.0–6.0, Fig. 58; weakly shagreened, ca. 2.0–5.0 puncture diameters on T2–T3); T4–T5 much more strongly shagreened throughout, with small punctures nearly contiguous to separated by 3.0 puncture diameters on basal three-fourths, minute punctures separated by 2.0–6.0 puncture diameters on apical fourth, with apical impunctate bands ca. 3.0–4.0 puncture diameters in length.

###### Structure:

Labial palpus four-segmented, second labial palpal segment ca. one-fourth longer than basal most segment. Mandible with condylar ridge about twice thickness of outer ridge, strongly converging apically (Fig. 5); apical margin with four distinct teeth, third separated from second and fourth by carina, margin of third tooth forming distinct V-shape with adjacent margin of second and forming weak concavity with margin of fourth, third tooth set back from second and fourth, very slightly directed inwards (Fig. 6); inner, ventral margin of mandible lacking distinct tooth, strongly diverging away from condylar ridge basally; mandible apically only slightly wider than narrowest point medially, first tooth subequal to, or very slightly longer than, second tooth, length between apical tips of second and fourth teeth 1.7 to nearly twice wider than apical tips of first and second teeth (Fig. 6). Clypeus with median truncation at apical margin linear to weakly concave, with truncation laterally weakly set off from remaining lateral margin of clypeus. F1 twice length of F2 or slightly more, remaining apical flagellar segments gradually increasing in length such that F10 about 1.2 times length of F1. Vertex behind lateral ocellus 2.5–3.0 OD in length. Genal width 1.0 to nearly 1.5 times that of compound eye in lateral view (wider dorsally). Preoccipital margin rounded, not carinate. Hypostomal carina moderately high, more or less level along length of head except reduced to obsolescence at angle, sometimes forming weak triangular projection posterior to angle. Malus forming pointed apical spine, this spine more or less a continuation of nearby edge of vellum. Foretarsal segments excluding basitarsal and apical-most segments with anterior lobes slightly longer than posterior. Midtarsal segments with anterior and posterior lobes of equal width, slightly swollen; hind tarsal segments not swollen. Hind tibial spurs slightly curved at apical tips, outer spur about a fifth shorter than inner. Hind basitarsal segment with lateral margins of outer surface parallel along most of length, converging apically.

#### Distribution.

In the Nearctic, Osmia laticeps is known from Yukon east to Nova Scotia, and as far south as Ontario and Michigan. In the Palearctic, Osmia laticeps is known from Germany northwest to Sweden and Finland, east to Latvia and northern Siberian Russia (Müller 2010).

#### Comments.

Osmia laticeps is oligoletic on Vaccinium (Ericaceae) (Nilsson 2009).

#### Material examined.

**CANADA: MANITOBA, Northern Region**, 12 June 1952 (1♂, Ottawa), 20 June 1930 (2♀, 1♂, Logan); **NOVA SCOTIA, Kings Co.**, 24 May 1932, apple (1♂, Ottawa); **ONTARIO, Kenora District**, 10 June 1964, Viola adunca (1♀, Ottawa); Ottawa, 22 May 1973 (1♂, Ottawa); **QUEBEC, Abitibi-Témiscamingue Region**, 24 May 1934 (1♀, Toronto); **Nord-du-Québec Region**, 9 June 1956 (1♀, Ottawa); **Bas-Saint-Laurent Region**, 22 June 1916 (2♀, Ottawa); **YUKON**, 22 May 1951 (1♂, Ottawa), 28 May 1951 (3♂, Ottawa), 2 June 1951 (2♀, Ottawa), 12 June 1960, 3500 ft (1♂, Ottawa), 17 July 1981 (1♀, Victoria); **RUSSIA: Siberia**, 11–15 July (1♀, Ottawa); **SWEDEN: Norrbotten Co.**, 6 July 1975 (1♀, Uppsala); **USA: MAINE**, 15 June 1982 (1♀, St. Charles); **MICHIGAN, Alger Co.**, 3–11 June 1982, sand pit (1♀, 1♂, New York), 28 June 1982, Vaccinium myrtilloides (1♀, St. Charles); **Marquette Co.**, 10 June 1985, Gaylussacia sp. (1♂, St. Charles), 18 June 1983 (1♀, St. Charles).

### Osmia Melanosmia maritima

Friese

Figs 7, 8 55, 57 59, 62 65

Osmia maritima Friese 1885: 85 [Lectotype female: Berlin]; Tkalců 1983: 152 [lectotype designation].

#### Diagnosis.

Osmia maritima is one of two currently known species of the xanthomelana species group in North America (species with more or less shining ventral area of the propodeal triangle, apically widened mandible in females, and distinctly swollen gonoforceps in males). Females of Osmia maritima are distinguished from the other North American xanthomelana species group member, Osmia nearctica, by characteristics of the mandible, outer hind tibial spur, and clypeus: the mandible has a third tooth that is recessed below a distinct carina between the second and fourth teeth (Fig. 8) (Osmia nearctica with the third tooth in the same plane as the second and fourth teeth and no carina, Fig. 10); the outer hind tibial spur is strongly curved apically (Osmia nearctica with outer hind tibial spur weakly curved apically), and the apical truncation of the clypeus is not distinctly set apart from the lateral apical margin of the clypeus, Fig. 55 (Osmia nearctica with the apical truncation forming a 90 degree angle with the lateral apical margin of the clypeus, Fig. 35). Females of Osmia maritima also have almost entirely black pubescence on the clypeus (significant amounts of light hairs throughout the clypeus in Osmia nearctica) and longer hair on the galea in dorsal view.

Males of Osmia maritima are distinguished from Osmia nearctica by their relatively long, sparse hairs on the lower surface of the flagellar segments (Osmia nearctica with these hairs microscopic) and weakly emarginate S2 (Osmia nearctica with S2 midapical margin not emarginate).

**Figures 64–70.**
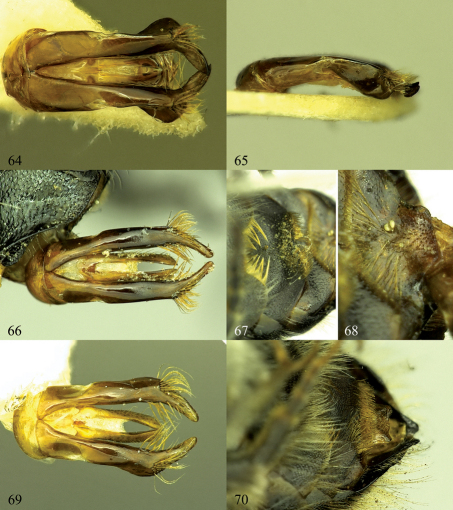
**64, 65.** Osmia maritima, male, genital capsule **64** Dorsal view **65** Lateral view. **66–68.** Osmia steinmanni, male paratype. **66.** Dorsal view of genital capsule **67** S3 and S4 **68** S5 and S6 **69, 70** Osmia svenssoni, male paratype. **69** Dorsal view of genital capsule **70** S4 and S6.

#### Distribution.

In the Nearctic, Osmia maritima is known only from the Northwest Territories and Alaska. In the Palearctic, Osmia maritima is known from the Netherlands, Germany, Denmark, Norway, Sweden, and Finland east to Mongolia and through Russia to Far Eastern Siberia (Müller 2010).

#### Comments.

We have not found any male specimens of Osmia maritima in the material of nearctic Osmia available to us. It is possible that once male specimens are discovered they may prove to be a distinct species from their palearctic relatives (if, as in Osmia aquilonaria, the novel diagnostic characters of the species are only found in the males); however, since a holarctic distribution is well established for other Osmia species (e.g., Osmia inermis and Osmia nigriventris), until proven otherwise we conservatively retain the name Osmia maritima for this species. Interestingly, there appear to be two female morphs of Osmia maritima. Specimens from Alaska and the Russian Far East share pale hair on the paraocular area and mesepisternum and scarcely sculptured apical areas on T2 and T3; females from the Northwest Territories and western Europe have dark hair on the paraocular area and mesepisternum and microsculptured apical areas of T2 and T3.

Osmia maritima from the Palearctic is known to be polylectic and nests in sandy soil with cells composed of chewed leaves and sand grains (Müller 2010 and references therein).

#### Material examined.

**CANADA: NORTHWEST TERRITORIES, Inuvik Region**, 17 June 1971 (1♀, Ottawa), 20–25 June 1971 (3♀, Ottawa), 28–30 June 1971 (1♀, Ottawa), 11 July 1948 (1♀, Ottawa); **NETHERLANDS: Terschelling**, 2 June 1969 (1♂, 1♀, Logan); **RUSSIA: Siberia**, 5 July 1992 (1♂, Davis), 12 July 1992 (1♀, Davis); **USA: ALASKA, Fairbanks North Star Borough**, 31 July 1985 (1♀, Davis); **Southeast Fairbanks Census Area**, 21 June 1984, Oxytropis campestris (3♀, Davis); **Yukon-Koyukuk Census Area**, 17 May 1991, Dodecatheon frigidum (1♀, Davis), 19 June 1992, Penstemon gormanii (1♀, Davis).

### Osmia Melanosmia nearctica

Rightmyer, Griswold, & Arduser sp. n.

urn:lsid:zoobank.org:act:E8E24354-14D3-463C-8505-F6E2002F401E

Figs 9, 10 33 51 

#### Diagnosis.

Osmia nearctica is one of two members of the xanthomelana species group in North America; characters to distinguish it from the other member of that group, Osmia maritima, are given under that species (see above).

In the Palearctic, Osmia nearctica is most similar to Osmia xanthomelana, but can be differentiated from that species by the following characters: In females, the propodeal triangle is shining but weakly shagreened throughout (Fig. 36) (Osmia xanthomelana with entirely polished, strongly shining lower half of the propodeal triangle), the outer hind tibial spur is only about half the length of the hind basitarsal segment (Osmia xanthomelana with outer hind tibial spur nearly three-fourths length of hind basitarsal segment), and the lower margin of the mandible has a distinct, translucent flange that curves away from the condylar ridge (Osmia xanthomelana with the lower margin of the mandible opaque, forming a ridge that is parallel to the condylar ridge). The hairs of the mesepisternum tend to be dark brown in Osmia nearctica, while in Osmia xanthomelana the hairs tend to be pale yellow to white, and the hairs of the hypostomal area tend to be denser in Osmia nearctica than in Osmia xanthomelana.

In males, the propodeal triangle is weakly shagreened throughout in Osmia nearctica (Fig. 41) (Osmia xanthomelana with entirely polished, strongly shining lower half of the propodeal triangle); the lower tooth of the mandible is only slightly longer than the upper tooth in Osmia nearctica (in Osmia xanthomelana the lower tooth of the mandible is much longer than the upper tooth and the entire apical margin of the mandible is conspicuously wider than the middle, approaching the look of male Acanthosmioides); T7 midapically has a shallower emargination in Osmia nearctica (Fig. 43) than in Osmia xanthomelana; the S6 midapical truncation is clearly emarginate in Osmia nearctica (Fig. 47) (Osmia xanthomelana with S6 truncation not emarginate); and the apical tip of the gonoforceps (apical to subapical swelling) is more rounded in Osmia nearctica (Figs 49, 50) (in Osmia xanthomelana the apical tip is more pointed). Osmia nearctica can be differentiated from Osmia maritima and Osmia alticola by the microscopic hairs on the underside of the flagellar segments (Osmia maritima and Osmia alticola with conspicuous hairs about half the width of the flagellar segments).

#### Description.

##### Female.

Figs 9, 10, 33–38. Total length: 9.0–11.5 mm; forewing length: 6.5–7.2 mm; length of lateral ocellus to preoccipital margin 0.6 mm; length of lateral ocellus to compound eye 0.7 mm.

###### Color:

Dark brown to brown-black, sometimes with reddish overtones especially on mouthparts, labrum, mandible, flagellar segments, legs, and apical margins of T1–T5. Wings moderately infuscate, more strongly infuscate in marginal cell and distal to cells.

###### Pubescence:

Clypeus below apical margin with lateral tuft of golden, medioposteriorly directed hairs. Brown, minutely branched hairs on most of body except as follows: white to yellow, minutely branched hairs interspersed with brown on outer surface of mandible, face excluding ventral third of clypeus and sometimes on vertex (and gena), and dorsal surfaces of T2, T5, T6; almost entirely white to yellow, minutely branched hairs on vertex (sometimes), mesoscutum, mesoscutellum, metanotum, dorsally on propodeum (excluding triangle), pronotal lobe, and dorsal surface of T1; brown, simple hairs interspersed with minutely branched hairs on most of body, except simple hairs lacking on dorsal mesosoma; simple hairs only (no branched hairs), golden on all tarsi and brown on scopa; brown, short, simple hairs evenly covering forewing. Galea and basal two labial palpal segments with hairs on lateral margins straight, 0.2–0.5 OD in length. Labrum with long hairs arranged in two curved, transverse rows, along subapical margin and approximately at midpoint, with additional fringe of shorter hairs at apical margin. Clypeus with hairs about as dense as on frons. Hypostomal area with hairs densely distributed across area, straight to weakly incurved at apical tips, 3.0–4.0 OD in length.

###### Punctation:

Head and mesosoma with punctures nearly contiguous, more or less round, and moderately impressed except as follows: labrum mostly impunctate except near fringes of hairs; clypeus with impunctate midapical truncation about length of F2 or little longer (Fig. 35); mesoscutum posterior to median longitudinal sulcus with punctures separated by up to a puncture diameter; mesepisternum with punctures less strongly impressed, nearly contiguous to separated by about half a puncture diameter; hypostomal area near angle and legs with punctures shallowly impressed, sometimes elongated into oval shape; tegula with punctures minute, sparser medially and posteriorly, separated by up to three or four puncture diameters; pronotum, metepisternum, metanotum, and lateral and posterior surfaces of propodeum with punctures very weakly impressed, with background integument weakly shagreened; propodeal triangle with dorsal fourth reticulate, lower three fourths shagreened, weakly shining (Fig. 36). T1 anterior surface weakly shagreened, shining, with scattered punctures at dorsolateral angle; T1–T3 dorsal surfaces very weakly shagreened, shining, excluding apical impunctate margins with small punctures nearly contiguous to separated by 1.0 puncture diameter (on T1, Fig. 37) to separated between 1.0 to 3.0 puncture diameters (on T3); apical impunctate bands 2.0–4.0 puncture diameters in length. T4–T5 dorsal surfaces shagreened, weakly shining, excluding apical impunctate bands with punctures nearly contiguous to separated by 2.0 puncture diameters; apical impunctate bands about 5.0–8.0 puncture diameters in length. T6 with punctures minute, nearly contiguous, mostly obscured beneath dense hairs.

###### Structure:

Labial palpus four-segmented, second labial palpal segment subequal to or ca. one-fourth longer than basal-most segment. Mandible with outer and condylar ridges of subequal thickness, parallel along length (Fig. 9); apical margin with four well-developed teeth, lacking carina separating third tooth from second and fourth, margin of third tooth forming distinct V-shape with adjacent margin of second and slightly smaller V-shape with adjacent margin of fourth, third tooth more or less on same plane as second and fourth (Fig 10); inner, ventral margin of mandible lacking distinct tooth, diverging away from condylar ridge basally; mandible apically widened (ca. 1.7 times wider than median width), first tooth longer than other teeth, length between apical tips of second and fourth teeth subequal to slightly wider than apical tips of first and second teeth (Fig. 10). Clypeus apical margin with distinct truncation on middle half, this truncation with lateral corner slightly produced, forming weak protuberance relative to apical margin of truncation and forming ca. 90 degree angle with apical margin of clypeus lateral to truncation (Fig. 35). F1 twice length of F2, remaining apical flagellar segments gradually increasing in length such that F10 subequal to F1 or little longer. Vertex behind lateral ocellus 2.0–2.5 OD in length. Genal width 1.5 to nearly 2.0 times that of compound eye in lateral view. Preoccipital margin rounded, not carinate. Hypostomal carina moderately high, highest at about midpoint of hypostomal area posterior to angle and forming distinct triangular projection at this point, tapering to low carina or near obsolescence at angle. Malus forming pointed apical spine. Foretarsal and midtarsal segments excluding basitarsal and apical-most segments with anterior lobes slightly longer than posterior; hind tarsal segments not swollen. Hind tibial spurs weakly curved, outer spur about a fifth shorter than inner. Hind basitarsal segment with lateral margins of outer surface parallel.

##### Male.

Figs 39–51. Total length: 8.6 mm (8.0–9.1 mm); forewing length: 6.0 mm (6.0–6.5 mm); length of lateral ocellus to preoccipital margin 0.5; length of lateral ocellus to compound eye 0.6 mm.

###### Color:

Black to dark brown, sometimes with reddish overtones especially on mouthparts, labrum, mandible, flagellar segments, legs, and apical margins of T1–T6 and S1–S3. Wings mostly clear except weakly infuscate along leading edge of forewing, especially along dorsal half of marginal cell.

###### Pubescence:

White to pale golden, minutely branched hairs on body except golden to pale golden, stouter, simple hairs on inner surfaces of tarsi, S4, and S6, and intermixed with white, branched hairs on mandible, lower gena, and outer surfaces of tarsi. Lample hairs lacking on dorsal mesosoma; simple hairs only (no branched hairs), golden on all tarsi and brown on scopa; brown, short, simple hairs evenly covering forewing. Galea and basal two labial palpal segments with hairs on lateral margins straight, 0.2–0.5 OD in length. Labrum with long hairs arranged in two curved, transverse rows, along subapical margin and approximately at midpoint, with additional fringe of shorter hairs at apical margin. Clypeus with hairs about as dense as on frons. Hypostomal area with hairs densely distributed across area, straight to weakly incurved at apical tips, 3.0–4.0 OD in length.

###### Punctation:

Head and mesosoma with punctures nearly contiguous, more or less round, and moderately impressed except as follows: labrum mostly impunctate except near fringes of hairs; clypeus with impunctate midapical truncation about length of F2 or little longer (Fig. 35); mesoscutum posterior to median longitudinal sulcus with punctures separated by up to a puncture diameter; mesepisternum with punctures less strongly impressed, nearly contiguous to separated by about half a puncture diameter; hypostomal area near angle and legs with punctures shallowly impressed, sometimes elongated into oval shape; tegula with punctures minute, sparser medially and posteriorly, separated by up to three or four puncture diameters; pronotum, metepisternum, metanotum, and lateral and posterior surfaces of propodeum with punctures very weakly impressed, with background integument weakly shagreened; propodeal triangle with dorsal fourth reticulate, lower three fourths shagreened, weakly shining (Fig. 36). T1 anterior surface weakly shagreened, shining, with scattered punctures at dorsolateral angle; T1–T3 dorsal surfaces very weakly shagreened, shining, excluding apical impunctate margins with small punctures nearly contiguous to separated by 1.0 puncture diameter (on T1, Fig. 37) to separated between 1.0 to 3.0 puncture diameters (on T3); apical impunctate bands 2.0–4.0 puncture diameters in length. T4–T5 dorsal surfaces shagreened, weakly shining, excluding apical impunctate bands with punctures nearly contiguous to separated by 2.0 puncture diameters; apical impunctate bands about 5.0–8.0 puncture diameters in length. T6 with punctures minute, nearly contiguous, mostly obscured beneath dense hairs.

###### Structure:

Labial palpus four-segmented, second labial palpal segment subequal to or ca. one-fourth longer than basal-most segment. Mandible with outer and condylar ridges of subequal thickness, parallel along length (Fig. 9); apical margin with four well-developed teeth, lacking carina separating third tooth from second and fourth, margin of third tooth forming distinct V-shape with adjacent margin of second and slightly smaller V-shape with adjacent margin of fourth, third tooth more or less on same plane as second and fourth (Fig 10); inner, ventral margin of mandible lacking distinct tooth, diverging awa y from condylar ridge basally; mandible apically widened (ca. 1.7 times wider than median width), first tooth longer than other teeth, length between apical tips of second and fourth teeth subequal to slightly wider than apical tips of first and second teeth (Fig. 10). Clypeus apical margin with distinct truncation on middle half, this truncation with lateral corner slightly produced, forming weak protuberance relative to apical margin of truncation and forming ca. 90 degree angle with apical margin of clypeus lateral to truncation (Fig. 35). F1 twice length of F2, remaining apical flagellar segments gradually increasing in length such that F10 subequal to F1 or little longer. Vertex behind lateral ocellus 2.0–2.5 OD in length. Genal width 1.5 to nearly 2.0 times that of compound eye in lateral view. Preoccipital margin rounded, not carinate. Hypostomal carina moderately high, highest at about midpoint of hypostomal area posterior to angle and forming distinct triangular projection at this point, tapering to low carina or near obsolescence at angle. Malus forming pointed apical spine. Foretarsal and midtarsal segments excluding basitarsal and apical-most segments with anterior lobes slightly longer than posterior; hind tarsal segments not swollen. Hind tibial spurs weakly curved, outer spur about a fifth shorter than inner. Hind basitarsal segment with lateral margins of outer surface parallel.

#### Distribution.

Canada from Yukon, the Northwest Territories, and Nunavut southeast to Ontario and Quebec.

#### Holotype male.

“[Canada] Norman Wells, N.W.T. [Northwest Territories], 13-VII-1949, W.R.M. Mason// Holotype male Osmia nearctica Rightmyer, Griswold, & Arduser” (Ottawa)

#### Paratypes.

**CANADA: MANITOBA, Winnipeg Capitol Region, Kettle Rapid**, near Winnipeg, 14 July 1917 (1♀, New York) ; **NORTHWEST TERRITORIES, Dehcho Region**, Hay River, 5 June 1951, P. R. Ehrlich (2♀, Ottawa); **Inuvik Region**, Reindeer Depot, MacKenzie Delta, 23 June 1948, W. J. Brown (1♀, Ottawa), 16 July 1948, J. R. Vockeroth (1♀, Ottawa); **Sahtu Region**, Norman Wells, 19 May 1953, C. D. Bird (1♀, Ottawa), 27 May 1953, C. D. Bird (1♀, Ottawa), 12 June 1949, W.R.M. Mason (2♀, Ottawa), 3 July 1949, W.R.M. Mason (1♀, Ottawa), 4 July 1949, W.R.M. Mason (1♀, Ottawa); **NUNAVUT, Kitikmeot Region**, Coppermine, 3 August 1951, S. D. Hicks (1♀, Ottawa); **ONTARIO, Thunder Bay District**, Black Sturgeon Lake, 13 June 1961 (1♂, Ottawa); **QUEBEC, Nord-du-Québec Region**, Rupert River, 10 July 1956, J. R. Lonsway (1♂, Ottawa); **YUKON**, Dempster Highway km 465, 15 July 1982, D. Wood (1♀, Ottawa).

#### Etymology.

The name “nearctica” is derived from the Greek arktikous, meaning northern or arctic, and is in reference to the known distribution of this species in northern regions of the New World (i.e., Canada).

### Osmia Melanosmia nigriventris

(Zetterstedt)

Figs 11, 12 53

Anthophora nigriventris Zetterstedt 1838: 465 [Syntype female: presumed lost (Tkalců 1995: 141)].
Osmia nigriventris  (Zetterstedt); Nylander 1848: 260.
Osmia baicalensis Radoszkowski 1867: 80 [Lectotype female: Berlin]; Friese 1909: 126 [synonymy with Osmia dimidiata Morawitz]; Zanden 1991: 353 [Lectotype designation, rejection of synonymy with Osmia dimidiata Morawitz, synonymy with Osmia nigriventris (Zetterstedt)].
Osmia frigida Smith 1853: 142 [Male and female syntype series: London]; Sandhouse 1939: 35 [synonymy].
Osmia hudsonica Cresson 1864: 21 [Holotype male: Philadelphia]; Sandhouse 1939: 35 [synonymy].
Osmia corticalis Gerstäcker 1869: 331 [Lectotype female: Berlin]; Thomson 1872: 244 [synonymy]; Tkalců 1995: 141 [Lectotype designation (but see Nilsson 2009: 50)].
Osmia (Melanosmia) nigriventris  (Zetterstedt); Schmiedeknecht 1885–1886: 79.
Osmia (Centrosmia) nigriventris  (Zetterstedt); Sinha 1958: 244; Sinha and Michener 1958: 284 [revision].
Osmia (Centrosmia) nigriventris frigida  Smith; Tkalců 1995: 141.

#### Diagnosis.

Females of this species are known by the swollen clypeal margin (Figs 11, 12) (approaching the extreme look found in Osmia bucephala, but unlike in that species, there is no metallic coloration in the integument of the meso- and metasomata). Males are known by the strongly reflexed apicolateral angles of T5 and T6 (Fig. 53). Unlike in Osmia bucephala, the midleg tarsal segments 2–4 are not modified or swollen, and S2 is unmodified (S2 of Osmia bucephala with a low tumescence bordered anteriorly and laterally by several rows of erect bristles).

#### Distribution.

In the Nearctic, Osmia nigriventris is known from Oregon, Idaho, Wyoming, and Michigan north to Yukon and the Northwest Territories, east across Canada to Ontario, Quebec, and Newfoundland. In the Palearctic, Osmia nigriventris is known from France, Italy, and Slovakia north to Norway, Sweden, and Finland and east to Mongolia, northern China, and through Russia to Far Eastern Siberia (Müller 2010).

#### Comments.

Osmia nigriventris is polylectic, with preference for Vaccinium (Ericaceae); it nests in old insect burrows in dead wood and constructs cell partitions and nest plugs with chewed leaves (Müller 2010 and references therein).

#### Material examined.

19 July 1955 (1♂, Ottawa), 28 July 1955 (2♀, Ottawa); **CANADA: ALBERTA, Alberta’s Rockies Region**, (1♀, Ottawa), 21 May 1915 (1♀, 3♂, Ottawa), 25 May 1922 (1♀, Ottawa), 3 July 1968, Dryas drummondii (1♀, Ottawa), 8 July 1968, Hedysarum sulphurescens (1♂, Ottawa), 23 August 1955, 4500 ft (1♀, Ottawa); **Central Alberta**, 8 June 1921 (1♂, Ottawa) **BRITISH COLUMBIA, Stikine District**, 6 June 1955, 2200 ft (1♀, Ottawa), 9 June 1955, 2200 ft (2♂, Ottawa), 26 July 1955, 2200 ft (1♀, Ottawa); **Columbia-Shuswap District**, 1 August 1950 (1♀, Ottawa), 1 August 1952, 6000 ft (1♀, Ottawa), 2 August 1952, 6000 ft (1♀, Ottawa); **Peace River District**, 11 June 1948 (1♂, Ottawa), **Thompson-Nicola District**, 8 August 1943 (1♀, Ottawa); **MANITOBA, Eastman Region**, June 1966 (1♀, Ottawa); **Northern Region**, 31 May 1949 (1♂, Ottawa), 3 June 1952 (1♂, Ottawa), 12 June 1952 (1♂, Ottawa), 19 June 1949 (1♂, Ottawa), 26 June 1950 (1♂, Ottawa), 29 June 1949 (1♀, Ottawa), 5 July 1950 (1♂, Ottawa), 10 July 1952 (1♀, Ottawa), 13 July 1937 (1♀, Ottawa), 15 July 1949 (1♂, Ottawa), 17 July 1937 (1♀, Ottawa); **NEWFOUNDLAND AND LABRADOR, Great Northern Peninsula**, 12 June 1951 (1♀, Ottawa); **NORTHWEST TERRITORIES, Dehcho Region**, 31 May 1969 (1♂, Ottawa), 5 June 1951 (2♂, Ottawa), 5 June 1969 (1♀, Ottawa); **Inuvik Region**, 13 June 1956 (1♀, 7♂, Ottawa), 16 June 1956 (1♀, 4♂, Ottawa), 18 June 1956 (2♀, Ottawa), 21 June 1910 (1♂, New York), 22 June 1948 (1♀, 1♂, Ottawa), 22 June 1956 (1♀, Ottawa), 25 June 1948 (1♂, Ottawa), 26 June 1948 (1♀, 1♂, Ottawa), 27 June 1948 (1♀, Ottawa), 28 June 1956 (1♀, Ottawa), 29 June 1956 (1♀, 1♂, Ottawa), 2 July 1948 (2♀, Ottawa), 3 July 1956 (3♀, Ottawa), 7 July 1948 (1♂, Ottawa), 10 July 1948 (1♀, Ottawa), 18 July 1948 (1♀, Ottawa), 25 July 1957 (1♀, Ottawa); **North Slave Region**, 9 July 1949 (1♀, Ottawa); **Sahtu Region**, 9 June 1949 (1♀, 2♂, Ottawa), 12 June 1949 (1♀, Ottawa), 18 June 1948 (1♂, Ottawa), 3 July 1972 (1♀, Ottawa); **ONTARIO**, 17 May 1962, Chamaedaphne sp. (1♂, Toronto); **Kawartha Lakes**, 24 May 1964, Taraxacum officinale (1♂, Ottawa), 10 June 1964, Taraxacum officinale (1♂, Ottawa); **QUEBEC, Nord-du-Québec Region**, 12 June–8 August 1987 (1♀, Ottawa), 19 June 1956 (1♀, Ottawa), 25 June 1956 (1♂, Ottawa), 25 July 1954 (1♀, Ottawa); **Côte-Nord Region**, 7 July 1948 (1♂, Ottawa); **YUKON**, 22 May 1951 (1♀, Ottawa), 28 May 1951 (1♀, 3♂, Ottawa), 29 May 1951 (1♀, 1♂, Ottawa), 1 June 1951 (1♀, Ottawa), 2 June 1951 (1♀, Ottawa), 5 June 1951 (1♂, Ottawa), 15 June 1960 (1♂, Ottawa), 15 June 1980 (1♂, Victoria), 17 June 1960, 3200 ft (2♀, Ottawa), 19 June 1960, 3000 ft (1♀, Ottawa), 22 June 1982 (1♀, 2♂, Victoria), 23–25 June 1980, 800 m (1♂, Logan), 27 June 1960, 3300 ft (1♀, Ottawa), 27 June 1984 (1♂, Victoria), 29 June 1984 (1♂, Victoria), 1 July 1949 (1♀, Victoria), 1–4 July 1973 (1♀, Ottawa), 1–5 July 1987, 720 m (1♂, Ottawa), 9 July 1983, 2300 ft (1♀, Victoria), 9 August 1981 (1♀, Victoria); **RUSSIA: Siberia**, 3 July 1992, Vaccinium vitis-idea (1♀, Davis); **USA: ALASKA, Fairbanks North Star Borough**, 5–13 May 2009 (1♀, Logan), 7 May 1982, Pulsatilla patens (1♂, Davis), 5 June 1987, Hedysarum mackenziei (1♀, Davis), 28 June 1987, Hedysarum sp. (1♀, Davis); **Southeast Fairbanks Census Area**, 22 May 1985, Arctostaphylos uva-ursi (1♀, Davis), 27 July 1982, Aster sibiricus (1♀, Davis); **Yukon-Koyukuk Census Area**, 30 June 1991, Hedysarum boreale (1♀, Davis); **IDAHO, Lemhi Co.**, 20 July 1963 (1♀, 1♂, Moscow); **MICHIGAN, Marquette Co.**, 18 May 1982, Amelanchier bartramiana (1♀, St. Charles), 19 May 1982, Amelanchier bartramiana (1♂, St. Charles); **MONTANA, Carbon Co.**, 22 June 1981, 6200 ft (1♀, Boulder), 11 July 1963, 5200 ft (1♀, Boulder); **OREGON, Deschutes Co.**, 19 July 1927, 5500 ft (1♀, Corvallis); **WASHINGTON, Okanogan Co.**, 2 July 2004 (1♀, Logan); **WYOMING, Fremont Co.**, 28 June 1990, 10400 ft, Arctostaphylos uva-ursi (1♂, Logan), 30 June 1990, 10500 ft (1♂, Logan); **Johnson Co.**, 22 July 1998 (1♀, Logan); **Teton Co.**, 14 July 1930 (1♂, New York).

## Supplementary Material

XML Treatment for Osmia Melanosmia aquilonaria

XML Treatment for Osmia Melanosmia inermis

XML Treatment for Osmia Melanosmia laticeps

XML Treatment for Osmia Melanosmia maritima

XML Treatment for Osmia Melanosmia nearctica

XML Treatment for Osmia Melanosmia nigriventris
